# Pancreatectomy with en bloc superior mesenteric vein resection and permanent mesocaval shunting for locally advanced pancreatic head cancer

**DOI:** 10.1093/jscr/rjaf624

**Published:** 2025-08-12

**Authors:** Takako Y Fujii, Takuya Kimura

**Affiliations:** Department of Liver Surgery, Yao Tokushukai General Hospital, 1-17 Wakakusa-cho, Yao City, Osaka 581-0011, Japan; Department of Liver Surgery, Yao Tokushukai General Hospital, 1-17 Wakakusa-cho, Yao City, Osaka 581-0011, Japan

**Keywords:** pancreatic head adenocarcinoma, pancreaticoduodenectomy, superior mesenteric vein invasion, mesocaval shunt

## Abstract

In pancreatic cancer (PC) with superior mesenteric vein (SMV) invasion, radical resection following neoadjuvant chemotherapy (NAC) is often feasible. While temporary mesocaval shunting (MCS) with subsequent portal vein (PV) reconstruction has been reported, PV reconstruction may be omitted in cases with sufficient collateral circulation. We report a case in which permanent MCS without PV reconstruction was employed to manage unexpected bowel congestion despite preoperative imaging suggesting adequate collateral flow. An elderly woman with borderline resectable uncinate PC and SMV invasion underwent pancreaticoduodenectomy with en bloc SMV resection following NAC. Intraoperatively, a 12-cm gap for reconstruction was noticed, and MCS was performed. The patient was discharged without liver dysfunction or hyperammonemia. This case highlights that permanent MCS may offer a salvage technique in select patients with SMV involvement, particularly when venous grafts are not available. It expands the surgical options in anatomically challenging cases.

## Introduction

In locally advanced pancreatic head cancer with superior mesenteric vein (SMV) invasion, radical resection can be achieved with neoadjuvant chemotherapy (NAC) followed by en bloc SMV resection. Two surgical strategies have been reported: one utilizes a temporary mesocaval shunting (MCS) during portal vein (PV) clamping to manage intraoperative bowel congestion, followed by portal vein reconstruction (PVR) using a venous graft (VG) [1]; the other omits PVR entirely in the presence of well-developed collateral circulation [2]. We report a case in which permanent MCS without PVR was employed to manage unexpected intraoperative bowel congestion, despite preoperative imaging suggesting that PVR could be omitted due to well-developed collateral circulation.

## Case report

**Figure 1:**
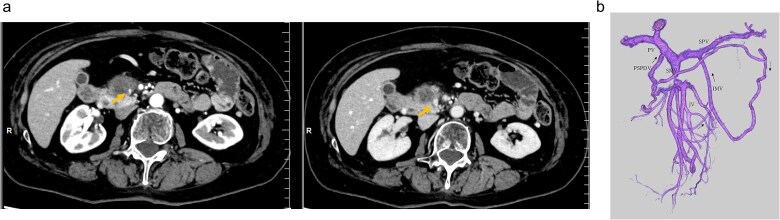
(a) Preoperative contrast-enhanced CT in the portal venous phase demonstrated a hypovascular tumor measuring 20 mm in the pancreatic uncinate process and mild improvement in SMV narrowing (arrow, pre-NAC in left image, post-NAC in right image). (b) Preoperative 3D imaging demonstrated that the jejunal veins were discontinued separately, and there are three collateral pathways, via posterior superior pancreaticoduodenal vein, inferior mesenteric vein through the right gastroepiploic vein, and stenotic SMV with intramesenteric anastomoses. SMV: Superior mesenteric vein, NAC: Neoadjuvant chemotherapy.

An elderly woman in her 80s presented to our hospital with prolonged epigastric pain. She was diagnosed with pancreatic cancer (PC) in the uncinate process, with severe stenosis of the SMV (Fig. 1a, left). She had previously undergone laparoscopic right hemicolectomy for cecal cancer 6 years earlier. The PC was considered borderline resectable without lymphadenopathy or evidence of distant metastasis, and she received three cycles of NAC with gemcitabine plus nab-paclitaxel (GnP). Although the SMV stenosis showed mild improvement after NAC (Fig. 1a, right), preoperative 3D imaging revealed that the jejunal veins drained separately and that prominent collateral pathways bypassing the SMV–PV axis had developed (Fig. 1b). We obtained fully informed consent, particularly regarding the potential procurement of a VG from her neck, and a pancreaticoduodenectomy was planned, preserving the collateral venous pathways and without SMV reconstruction.

**Figure 2:**
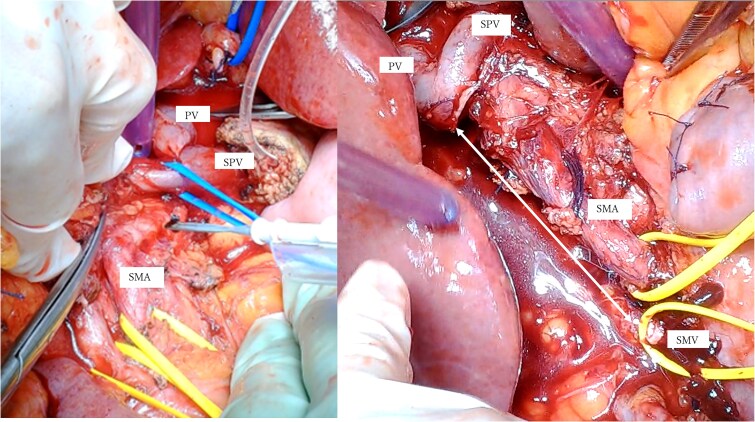
(a) Intraoperative photographs: Hemicircumferential dissection of the SMA nerve plexus, followed by en bloc SMV resection. (b) The SMV was transected proximal to its junction with the splenic vein. The SMA and SMV are encircled with vessel loops. The photograph illustrates the considerable gap between the SMV transection site and the jejunal branch stump (double-headed arrow). SMA: Superior mesenteric artery, SMV: Superior mesenteric vein.

**Figure 3:**
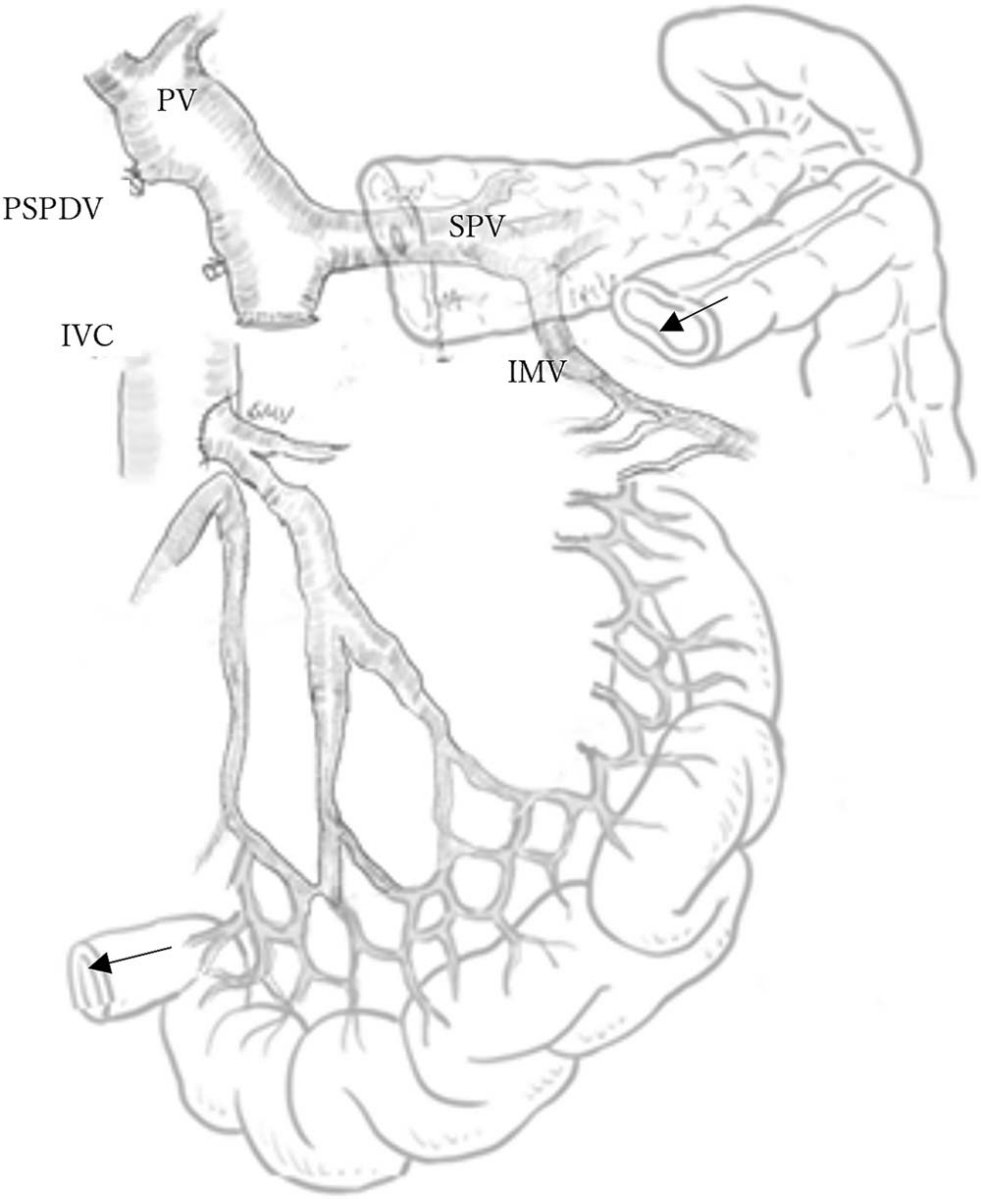
Schematic of mesocaval shunt and hepatic blood flow via collateral pathways. The arrows indicate the splenic flexure of the colon and the terminal ileum, which are scheduled for anastomosis. PV: Portal vein, SPV: Splenic vein, PSPDV: Posterior superior pancreaticoduodenal vein, IVC: Inferior vena cava, IMV: Inferior mesenteric vein.

A mesenteric approach was first undertaken, and both the SMV and SMA were encircled at the root of the small bowel mesentery. After pancreatic transection, the hemicircumferential nerve plexus dissection of the SMA was performed (Fig. 2, left). Following encirclement proximal to its confluence with the SPV, the SMV was transected using an Endo GIA™ stapler and resected en bloc during a classic Whipple procedure. Although no congestion was observed during test clamping of the stenotic SMV, significant jejunal congestion was noted after resection. There was a considerable gap of ⁓12 cm between the stump of the proximal JV and the PV (Fig. 2, right), such that a VG from the neck alone could not reach. As a result, a shunt was created between a jejunal branch of the SMV and the inferior vena cava (IVC) (Fig. 3). Reconstruction was performed using a modified Child procedure. The transverse colon was resected up to the splenic flexure, and ileocolic re-anastomosis was conducted at a well-perfused site (Fig. 3). Operative time was 463 min and estimated blood loss was 400 cc.

**Figure 4:**
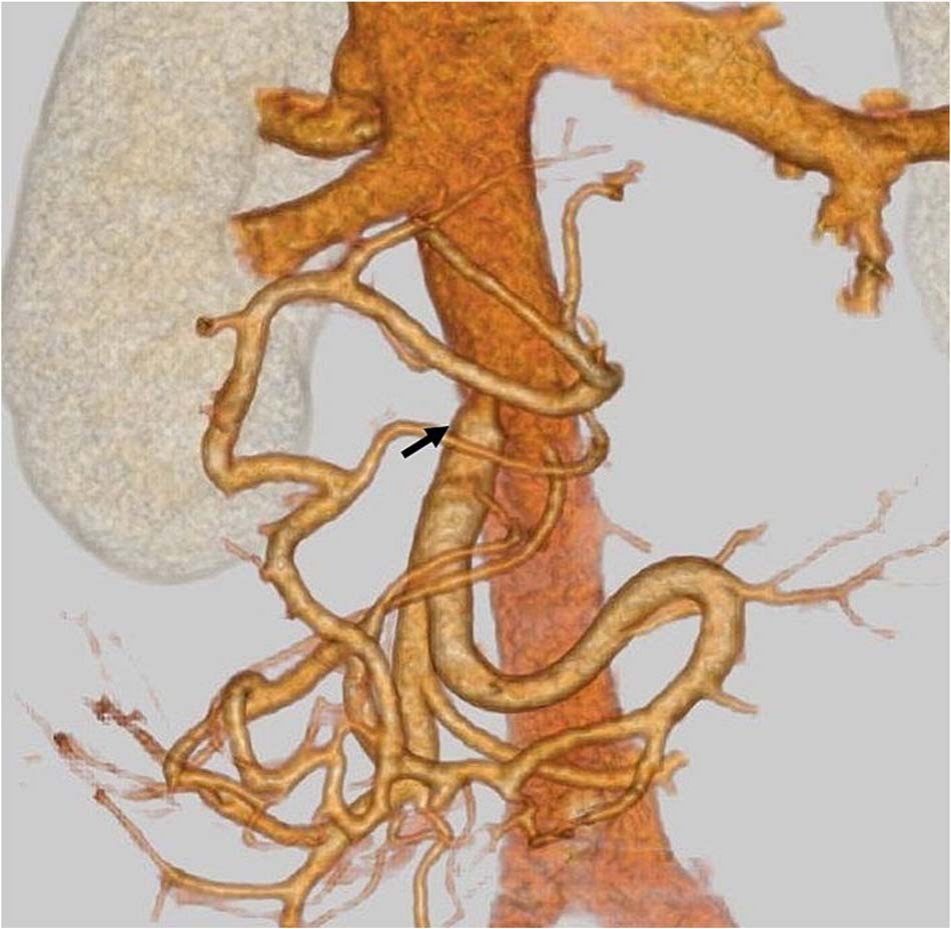
Postoperative contrast-enhanced CT: An arrow indicates patent mesocaval shunt (on postoperative day 14).

Postoperatively, the patient developed a pancreatic fistula classified as Clavien–Dindo Grade II [3]. Contrast-enhanced CT confirmed hepatic perfusion via collateral venous pathways through the SPV, and demonstrated patency of the SMV–IVC shunt (Fig. 4). The shunt was confirmed to be patent on serial imaging during the 4-month postoperative period. The patient hasn’t developed hyperammonemia or liver failure, and she was discharged on postoperative day 30th. Pathological examination shows ypT3 (22 mm), ypPV1 (PVp), ypA0, R0, ypN0 (0/11), pStageIIA.

## Discussion

Surgical resection remains the only potentially curative treatment for PC. Multimodal treatment approaches incorporating NAC, such as gemcitabine plus S-1, GnP, and FOLFIRINOX—have demonstrated strong antitumor efficacy in recent years. According to the NCCN Guidelines Version 1.2020 [4], it can be understood as implying that unreconstructible PV/SMV due to tumor involvement or anatomical complexity is classified as unresectable PC. When evaluating the anatomical limit for feasible anastomosis, a multicenter study from Japanese and Korean facilities [5] reported the feasibility of sacrificing branch of JV followed by direct anastomosis between the JV to the PV/SMV. In their cases requiring an interposition graft, all cases used allogeneic VG, suggesting the need for longer grafts when resecting PV/SMV with JV. In general, when venous invasion is extensive, such as in pancreatic uncinate cancer where the involved SMV, the segment may reach 7–8 cm [6] — or when multiple venous anastomoses are required, surgical planning becomes challenging in facilities without access to allogeneic VGs.

In terms of prognosis, the same study showed no significant differences in overall or recurrence-free survival regardless of the extent of JV resection [5]. Achieving R0 resection and administering adjuvant therapy were identified as independent prognostic factors. Therefore, careful preoperative patient selection is crucial to identify candidates who are likely to tolerate adjuvant treatment. The most common type of recurrence after resecting PV/SMV is reported as liver metastasis [7], possibly due to the spread via the portal circulation, especially when the tumor invades venous structures deeply.

The utility of a temporary MCS has been reported to help prevent bowel congestion caused by portal clamping during surgery for cases with PV-SMV involvement [1]. In select cases with well-developed collateral circulation, pancreaticoduodenectomy without PVR has also been described [2]. Sugitani *et al.* [2] proposed that preservation of the colic venous arcade and the porto-mesenterico-splenic confluence may allow for safe omission of SMV reconstruction. Our patient had previously undergone an ileocecal resection, in which the right colic venous system was disrupted. Although we initially planned SMV non-reconstruction, considering the SMV had already been chronically narrowed by tumor invasion and multiple collateral veins had developed from the dilated jejunal mesentery, suggesting compensatory drainage via the inferior mesenteric vein as reported in the literature [6], significant venous congestion was observed intraoperatively. This was likely due to insufficient collateral flow and residual dependence on the stenotic SMV for mesenteric drainage. Accordingly, MCS was promptly constructed to relieve venous congestion following SMV disconnection.

When intraoperative findings such as bowel congestion or unexpected complications necessitate venous reconstruction, particularly in cases like ours, where prior hemicolectomy resulted in a shortened mesentery limiting mobilization, and harvesting multiple autologous veins was not feasible, a permanent MCS may be considered. Given these anatomic and technical limitations, and in the absence of liver discoloration or ischemia, the shunt was deemed the most feasible and safe option to decompress intestinal venous outflow while preserving hepatic perfusion via the existing collateral pathways. MCS without PVR may present a surgical option to technically expand the resectability in anatomically complex or surgically challenging cases.

The Diverts portal blood directly into the systemic circulation draws upon surgical techniques developed in the field of transplantation surgery. While traditionally considered to carry a risk of hepatic encephalopathy and liver function deterioration, similar procedures are used in pancreas [8] and small bowel transplantation [9]. The IVC is utilized not only as a single route of the intestinal drainage but also as an auxiliary route to decompress excessive portal inflow in the small-for-size liver graft [10]. Randomized clinical data in small bowel transplantation have shown no significant difference in outcomes between anastomosis to the IVC and the SMV [9]. In patients with preserved hepatic reserve, the risk of hepatic encephalopathy is generally low, owing to the liver’s second-pass metabolic capacity.

## Conclusion

This case highlights that permanent MCS without PVR can be a surgical option in selected patients with pancreatic head cancer and SMV involvement, particularly when collateral circulation is present but proves insufficient intraoperatively. This approach may offer a salvage strategy for managing bowel congestion when conventional venous reconstruction is not feasible due to a long resection gap and/or limited VG availability.
